# Supplementary material for: The NDE1 genomic locus can affect treatment of psychiatric illness through gene expression changes related to microRNA-484
Source: Open Biol. 2017 Nov 15;7(11):170153. doi: 10.1098/rsob.170153 (PMC5717342; doi:10.1098/rsob.170153)
Supplement: Supplementary Tables and Figures [file rsob170153supp1.pdf]

Supplementary Information

The *NDE1* genomic locus can affect treatment of psychiatric illness through gene expression changes related to MicroRNA-484

Nicholas J. Bradshaw[1,2], Liisa Ukkola-Vuoti[3,5,6], Maiju Pankakoski[3], Amanda B. Zheutlin[8], Alfredo Ortega-Alonso[3,5], Minna Torniainen-Holm[3,5], Vishal Sinha[3,5,6], Sebastian Therman[3], Tiina Paunio[4,9], Jaana Suvisaari[3], Jouko Lönnqvist[3,9], Tyrone D. Cannon[8], Jari Haukka[3,7], William Hennah[3,5,6]*

[1]Department of Neuropathology, Heinrich Heine University, 40225 Düsseldorf, Germany
[2]Department of Biotechnology, University of Rijeka, 51000 Rijeka, Croatia
[3]Mental Health Unit, and [4]Genomics and Biomarkers Unit, Department of Health, National Institute for Health and Welfare, 00271, Helsinki, Finland
[5]Institute for Molecular Medicine Finland FIMM, [6]Medicum, and [7]Department of Public Health, Clinicum, University of Helsinki, 00014 Helsinki, Finland
[8]Department of Psychology, Yale University, New Haven, CT 06520, USA
[9]Department of Psychiatry, University of Helsinki and Helsinki University Hospital, 00014 Helsinki, Finland

*Corresponding Author:
William Hennah PhD
Institute for Molecular Medicine Finland FIMM, P.O. Box 20, FI-00014 University of Helsinki, Finland
Email: william.hennah@helsinki.fi

FIGURES

Figure S1: Graphical representation of the genetic effect of the *NDE1* rs2242549 SNP on the gene expression levels in the discovery cohort of a) the largest GTEx replicated positive effect *RAB24* (ILMN_2379718) (recessive model [GG and GT vs TT] p=3.5x10^{-6}) b) the largest GTEx replicated negative effect *SCNN1D* (ILMN_1754757) (recessive model [GG and GT vs TT] p=6.75x10^{-4}), c) the effect on *TRIOBP* (ILMN_1735788) (recessive model [GG and GT vs TT] p=2.99x10^{-6}) which replicates from our previous study but not in the GTEx database, and d) the non-significant effect on *NDE1* (ILMN_1739805) which is highly significant in the GTEx database (p=2.3x10^{-10}).

a)

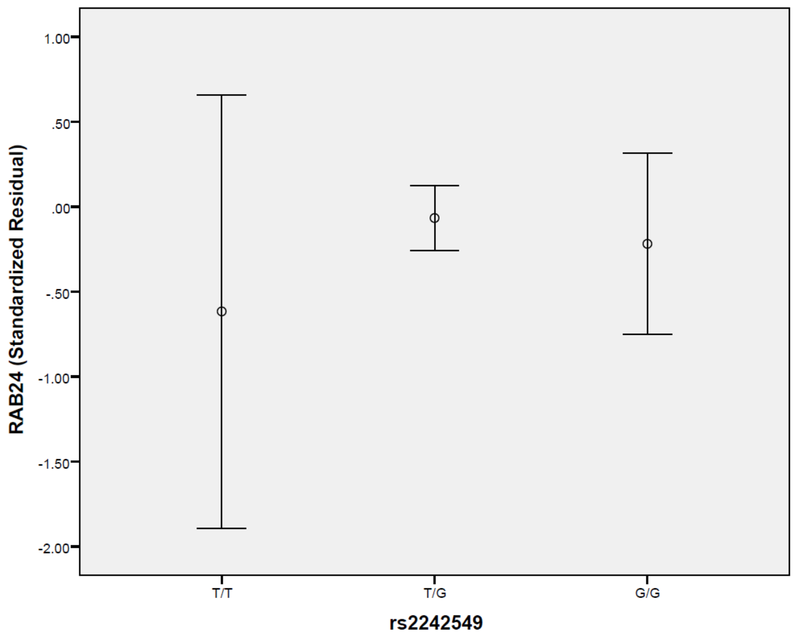

b)

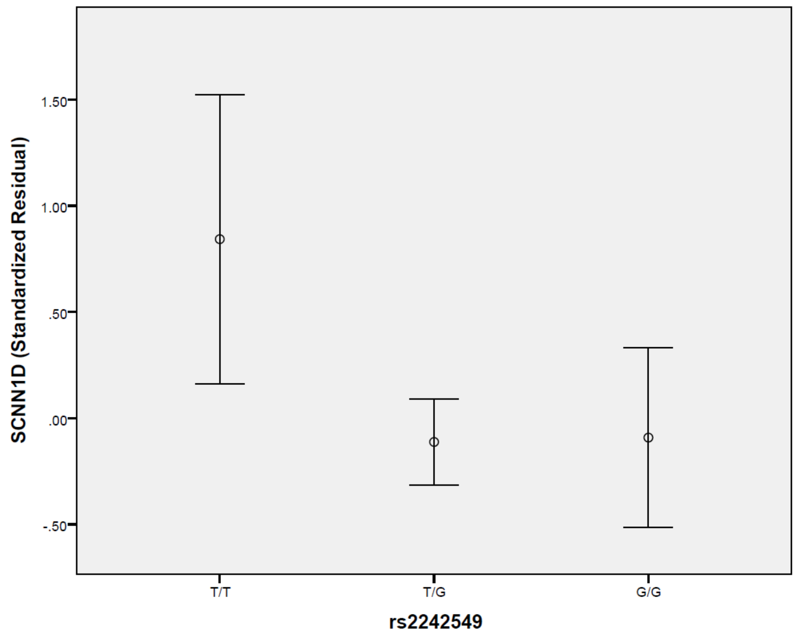

c)

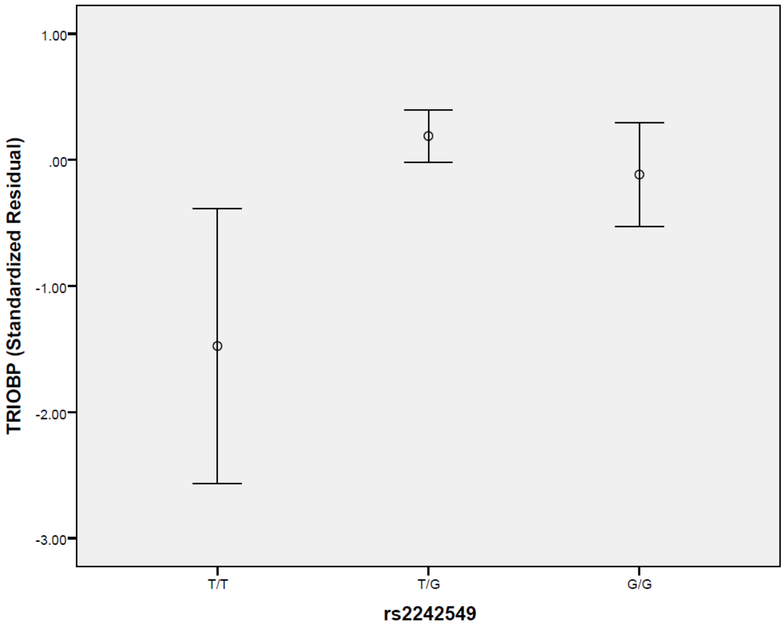
1.00
.00
-1.00
-2.00
-3.00
TRIOBP (Standardized Residual)
T/T
T/G
G/G
rs2242549

d)

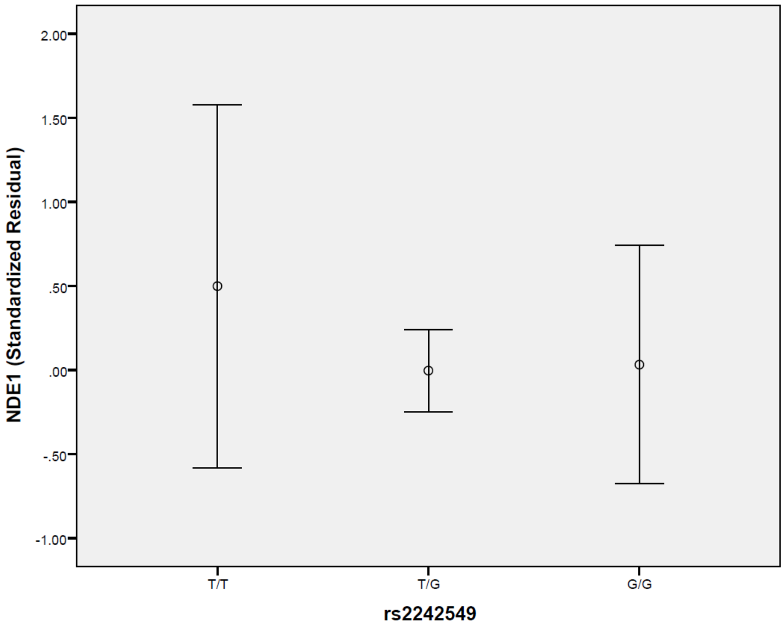
2.00
1.50
1.00
.50
.00
-.50
-1.00
NDE1 (Standardized Residual)
T/T
T/G
G/G
rs2242549

Figure S2: Power estimation of the discovery cohort to study the role of the *NDE1* SNP rs2242549 with the gene expression data (n=39) with two effect sizes. For both estimations, the observed 90th percentile of the standard deviation for all genes from our data (σ= 0.527), and the value of estimated proportion of non-differentially expressed probes based on qvalue-calculations (pi0 = 0.485) were used. The effect size of the estimation a) was the maximum observed in our discovery cohort (Δ=0.52), and b) the minimum effect size able to give the power of 80 % (Δ= 0.250). For probes with smaller standard deviations, the power is underestimated.

a)

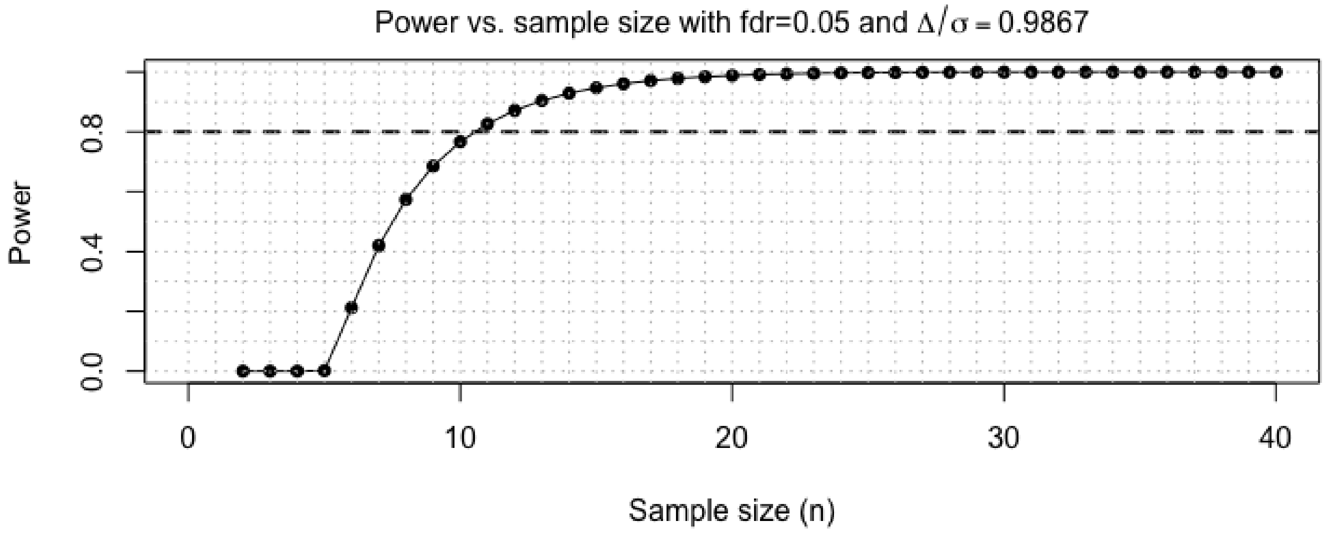

b)

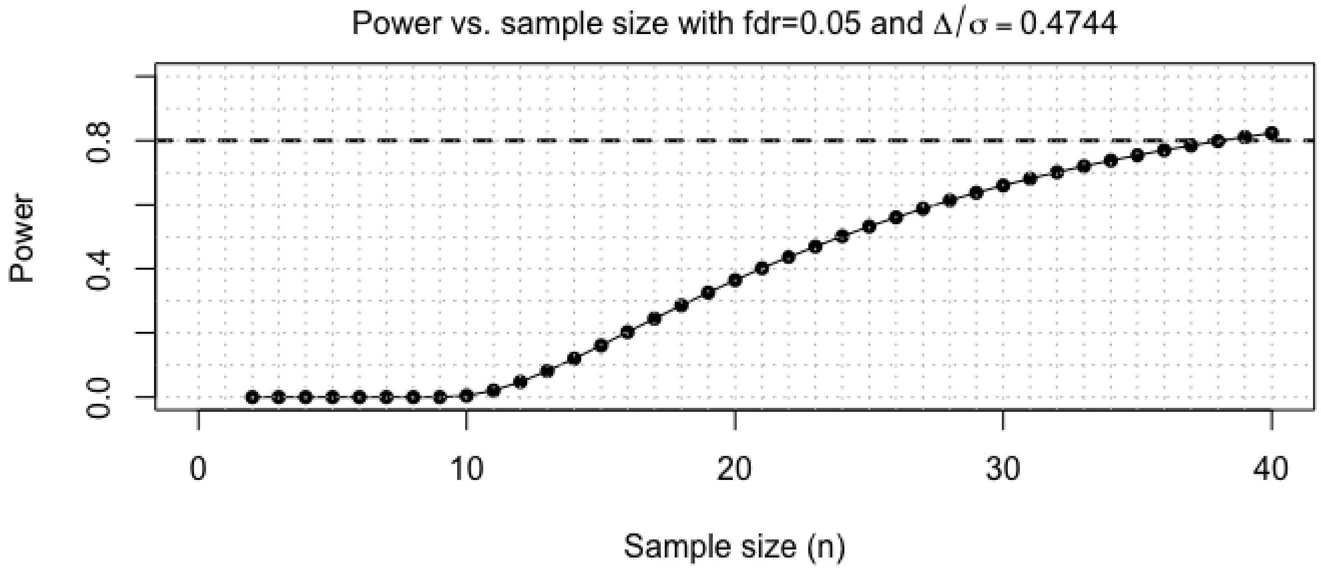

Figure S3:
The linkage disequilibrium (LD) structure of the 7 SNPs previously genotyped on the larger Finnish family cohort for schizophrenia as illustrated by the Haploview (R1) program. a) Shows the LD as defined by D' and therefore represents the haplotypic structure of the region, whereas b) shows the R^2 LD and therefore represents which SNPs are directly correlated with each other and thus. Block structures are shown based on the respective prediction tools implemented in Haploview (58), "solid spine of LD" for D' blocks and "confidence intervals" (59) for R^2 blocks.

a)

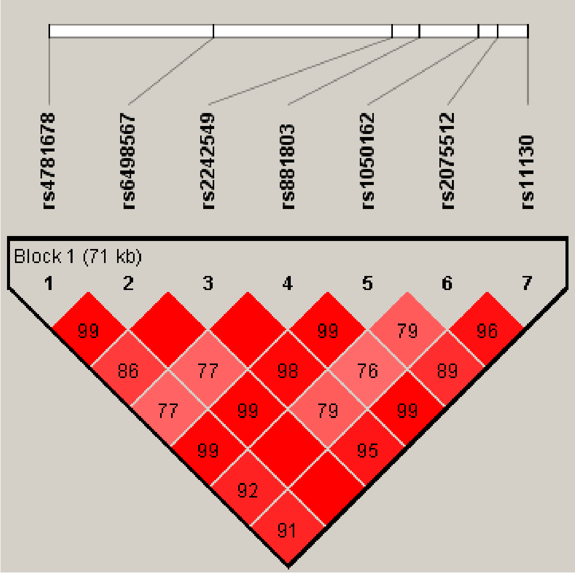

b)

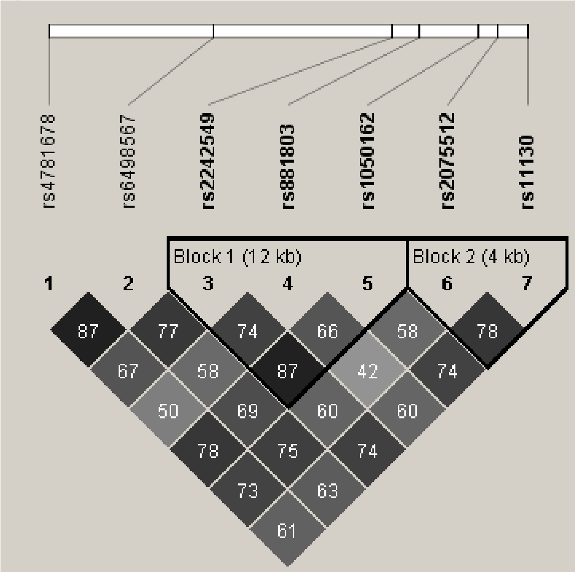

Figure S4:
Graphical representation of the gender by genetic interaction effect on medication groupings. Significance values can be found in Table 2.

All psychoactive drugs metabolised by CYP2C19 (Amitriptyline, Citalopram, Diazepam, Escitalopram, Fluoxetine, Mianserin, Sertraline)

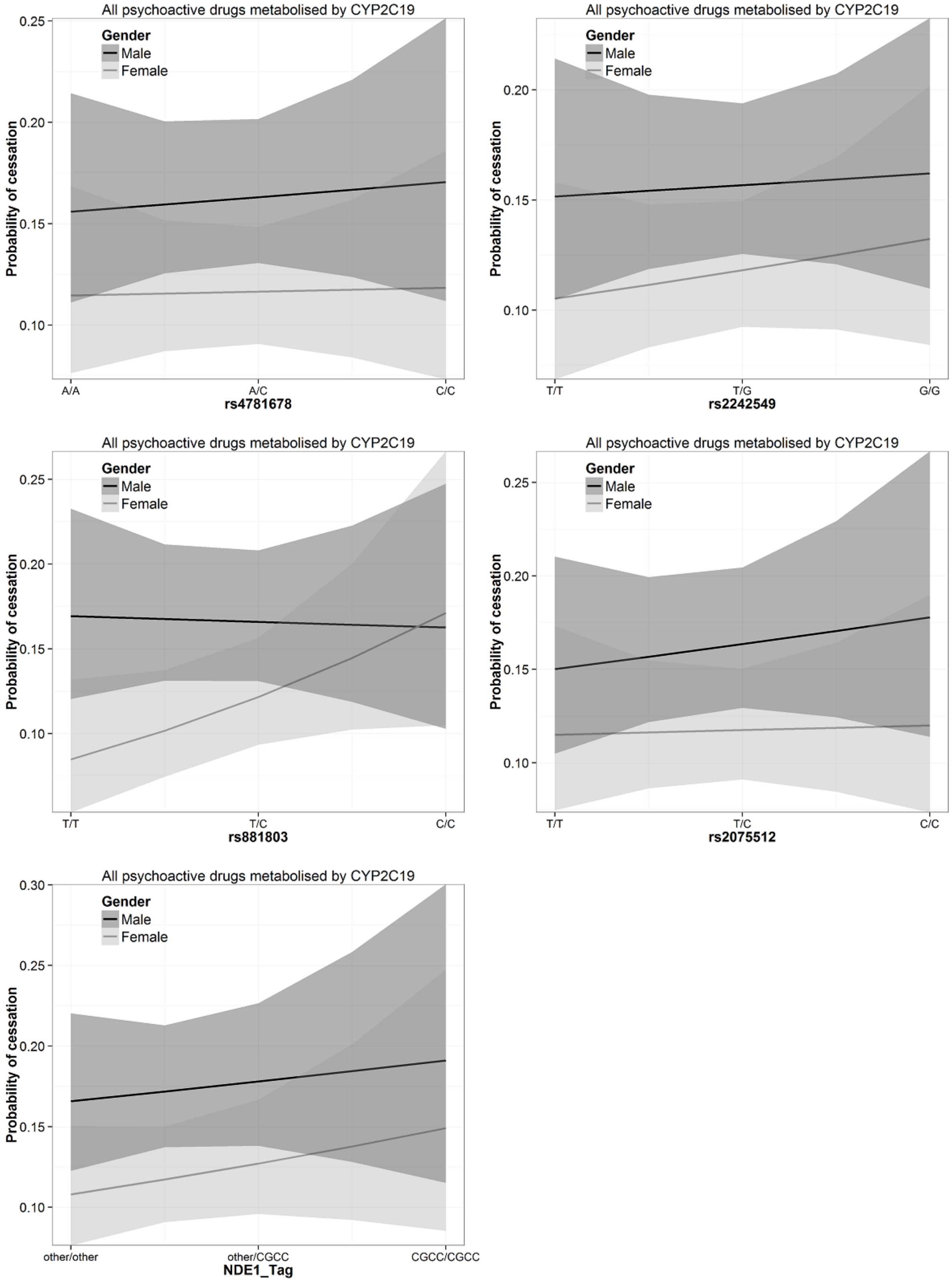

SSRIs (Citalopram, Escitalopram, Fluvoxamine, Fluoxetine, Paroxetine, Sertraline)

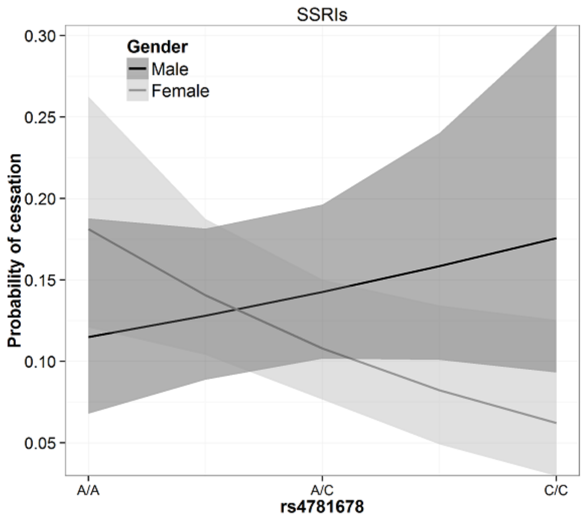

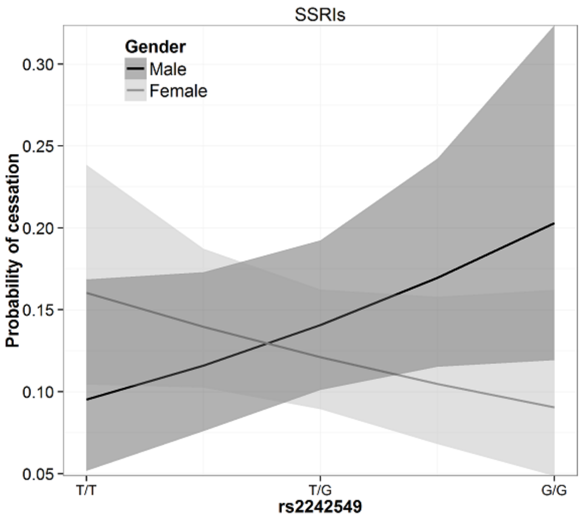

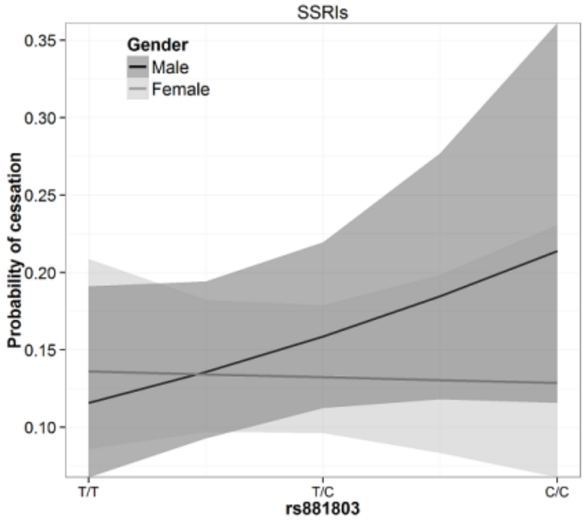

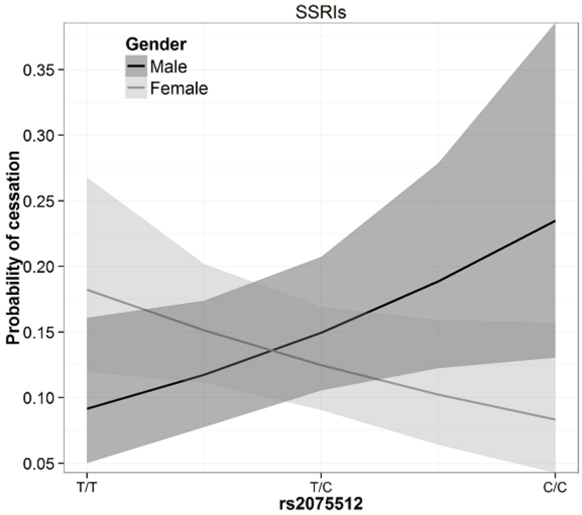

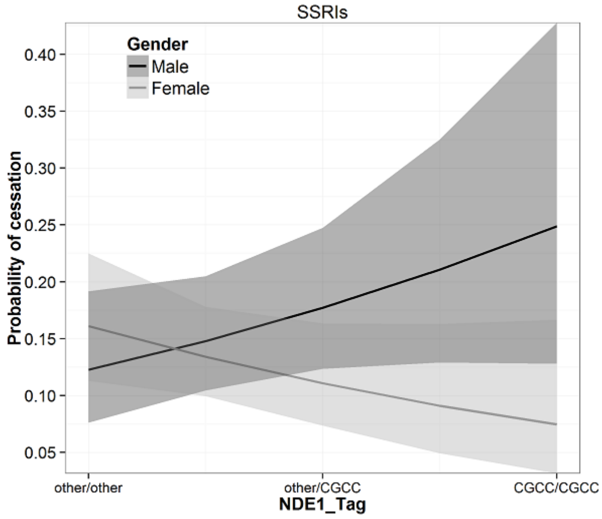

SSRIs metabolised by CYP2C19 (Citalopram, Escitalopram, Fluoxetine)

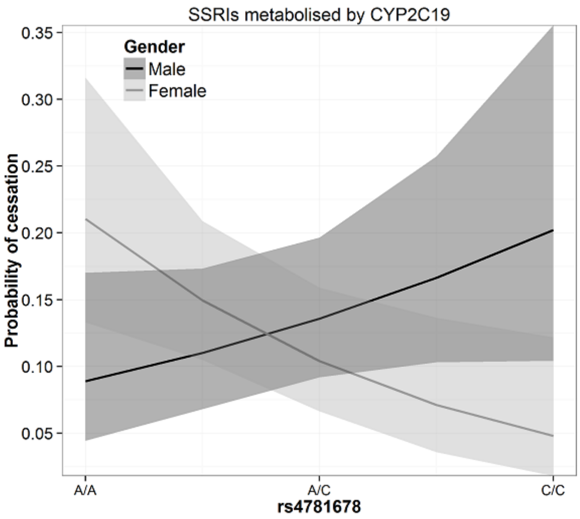

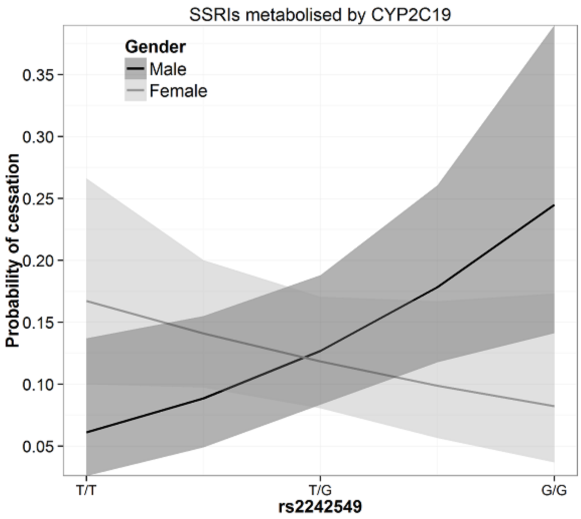

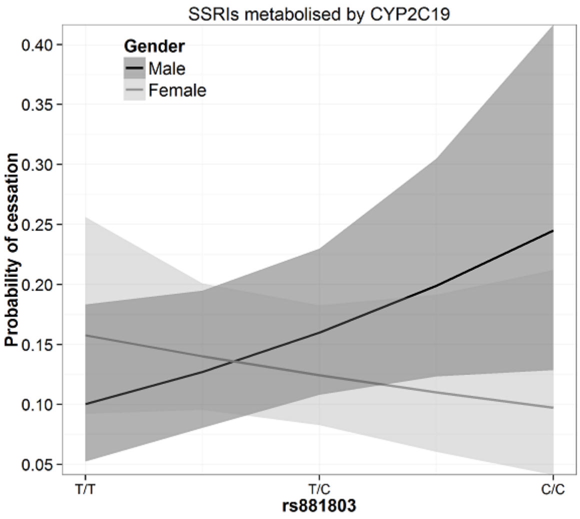

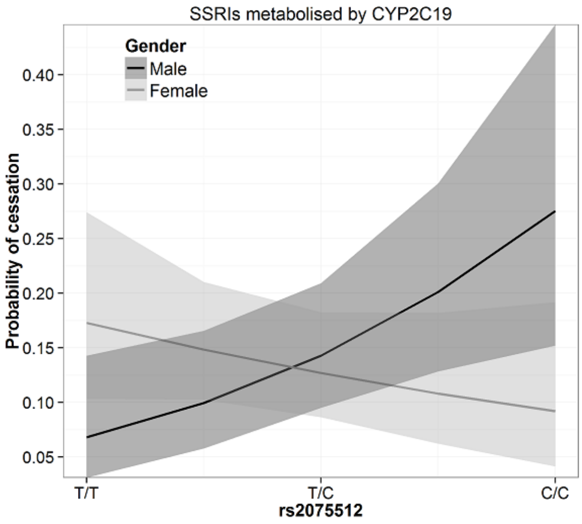

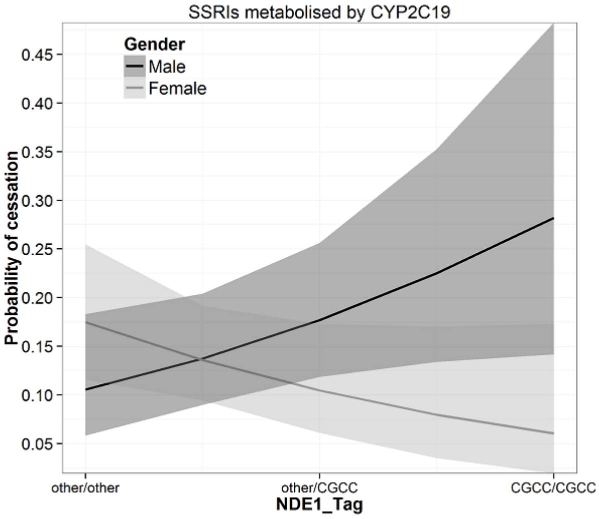

SSRIs not metabolised by CYP2C19 (Fluvoxamine, Paroxetine, Sertraline)

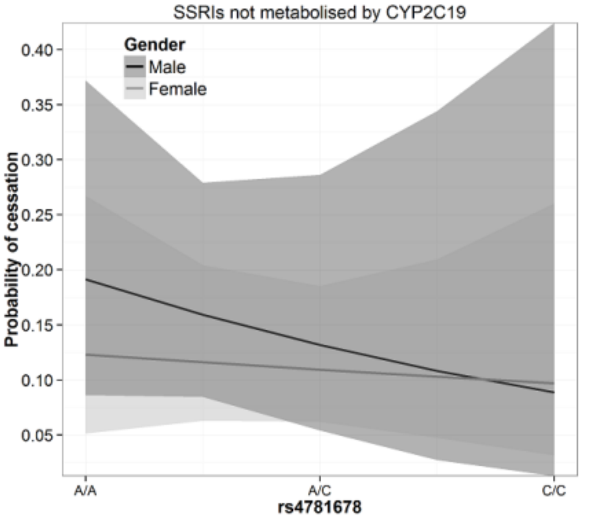

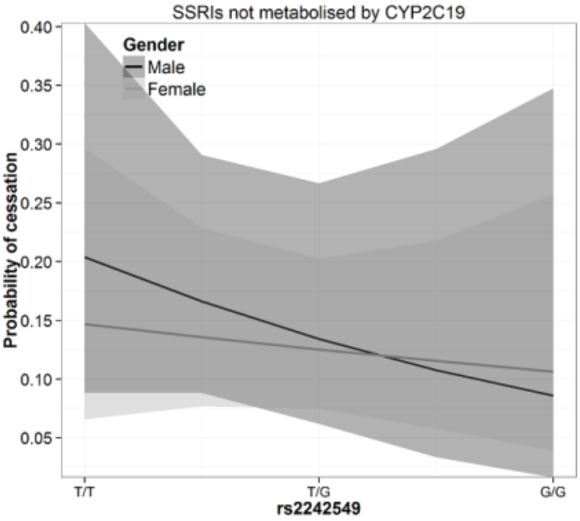

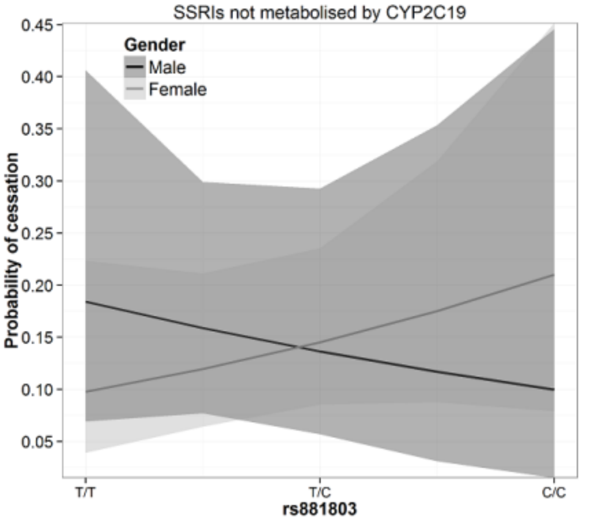

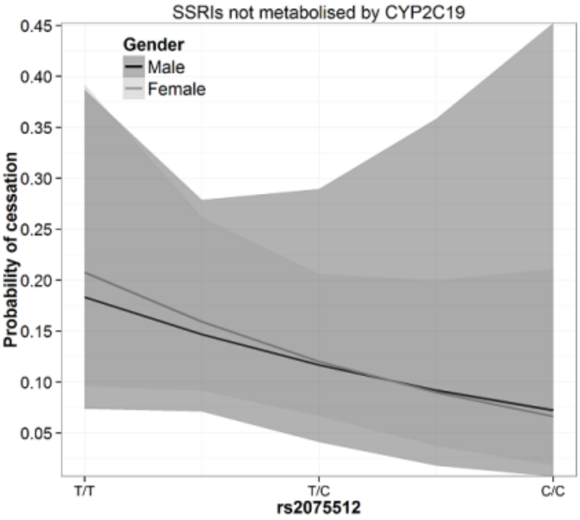

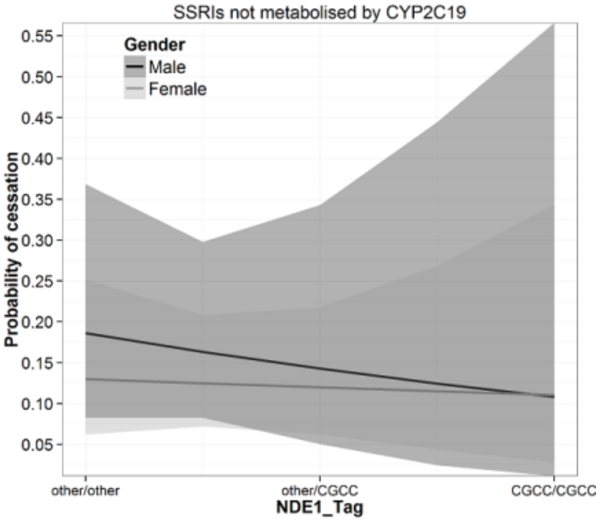

Non-SSRIs metabolised by CYP2C19 (Amitriptyline, Diazepam, Mianserin)

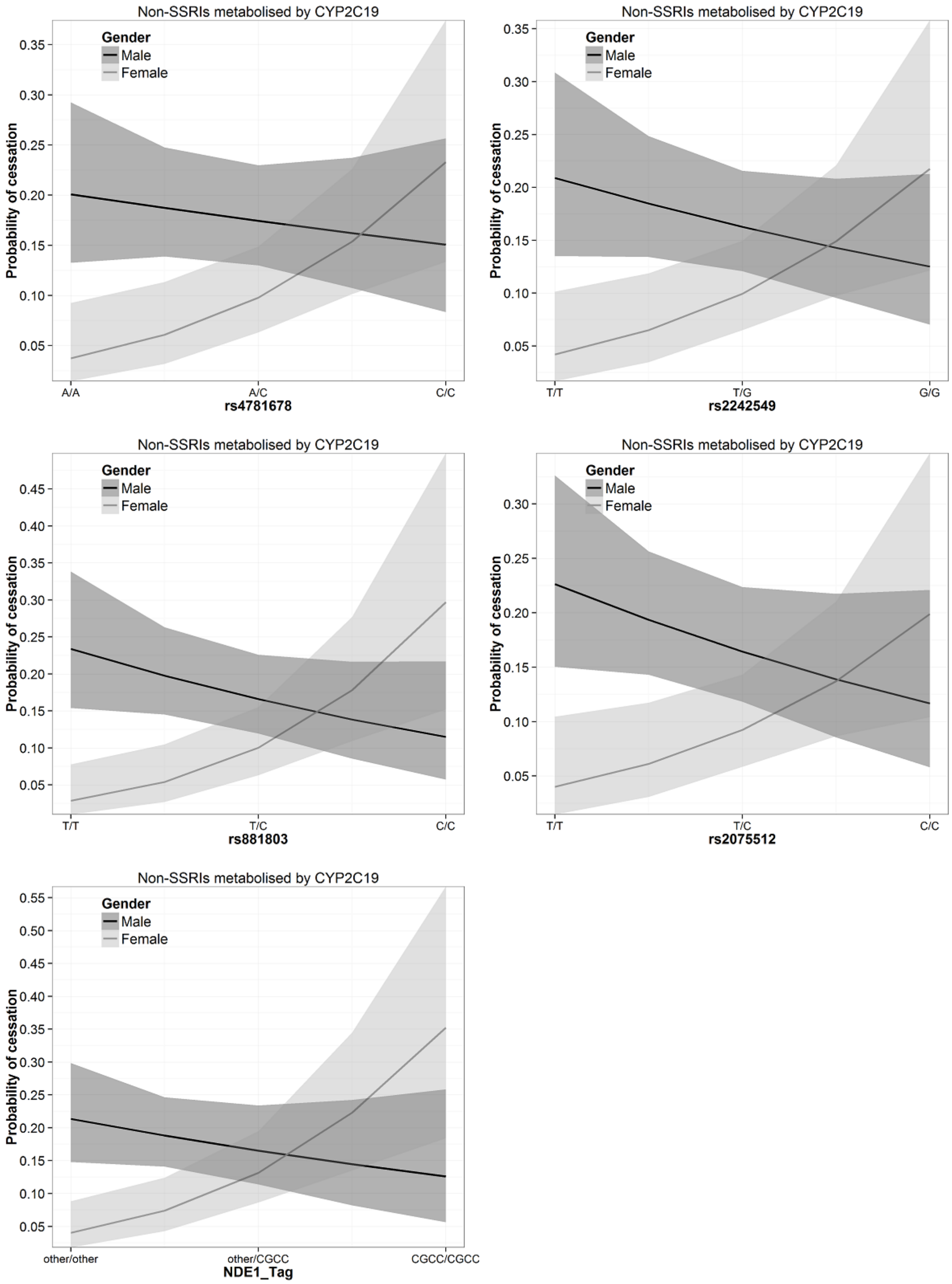

TABLES

Table S1: Results of the genome wide gene expression analysis and replication. Sheet 1 (Table S1 a) p-value <0.05) lists all probes that were significantly altered for each of the five DISC1 network variants tested at the p-value threshold of p<0.05. Table includes effect size (β), standard error, t value and p-value for the three cohorts tested. In addition it lists those genes replicated from our previous study, and the q-value for the study in the family cohort. Furthermore, if the probe is to a gene predicted to be targeted by mir-484 by six or more programs as annotated by miRWalk, then in the mir-484 target column the word "Target" appears. Sheet 2 (Table S1 b) q-value <0.05) list all the same properties as in Sheet 1, but restricted to those probes with a q≤0.05 after applying the False Discovery Rate.

Separate Excel File: Table S1.xlsx

Table S2: Results of the association analysis between individual psychoactive medications and the variants studied. a) p-values for the additive model controlling for gender, b) p-values for the additive model in interaction with gender, c) Odds ratios (and 95% CI) for the interaction terms that were significant at the uncorrected p-value ≤ 0.05 level. P-values, and their respective ORs, below 0.0021 are below the Bonferroni correction threshold for the 24 medications tested.

a)

	DISC1 **rs821616**	***NDE1*** **rs4781678**	***NDE1*** **rs2242549**	***NDE1*** **rs881803**	***NDE1*** **rs2075512**	***NDE1*** **Tag Haplotype**	***PDE4B*** **rs7412571**
**Tramadol**	0.96	0.93	0.43	0.36	0.42	0.57	0.041
**Paracetamol**	0.64	0.53	0.79	0.45	0.82	0.35	0.081
**Biperiden**	0.77	0.37	0.54	0.24	0.62	0.20	0.78
**Chlorpromazine**	0.50	0.79	0.095	0.35	0.15	0.67	0.054
**Levomepromazine**	0.98	**0.0009**	0.056	0.019	0.0022	0.018	0.17
**Perphenazine**	0.78	0.54	0.33	0.52	0.71	0.48	0.19
**Thioridazine**	0.048	0.12	0.10	0.16	0.83	0.31	0.94
**Haloperidol**	0.82	0.69	0.66	0.68	0.64	0.98	0.061
**Chlorprothixene**	0.74	0.35	0.60	0.20	0.32	0.34	0.65
**Zuclopenthixol**	0.60	0.31	0.15	0.85	0.29	0.80	0.24
**Clozapine**	0.50	0.79	0.91	0.93	0.66	0.64	0.80
**Olanzapine**	0.78	0.38	0.10	0.27	0.23	0.64	0.32
**Quetiapine**	0.02	0.30	0.58	0.76	0.89	0.92	0.74
**Risperidone**	0.23	0.068	0.49	0.63	0.081	0.061	0.70
**Diazepam**	0.14	0.20	0.63	0.64	0.68	0.28	0.0083
**Oxazepam**	0.67	0.57	0.71	0.90	0.10	0.19	0.45
**Temazepam**	0.38	0.17	0.017	0.15	0.77	1.00	0.041
**Zopiclone**	0.43	0.66	0.57	0.83	0.98	0.67	0.85
**Amitriptyline**	0.68	0.057	0.29	0.27	0.52	0.034	0.75
**Fluoxetine**	0.56	0.18	0.88	0.89	0.67	0.66	0.35
**Citalopram**	0.79	0.86	0.48	0.55	0.44	0.81	0.94
**Sertraline**	0.98	0.72	0.35	0.43	0.32	0.92	0.77
**Mianserin**	0.90	0.16	0.42	0.36	0.16	0.34	0.68
**Mirtazapine**	0.64	0.20	0.35	0.69	0.29	0.65	0.81

p-values that are below the Bonferroni threshold are in bold.

b)

	DISC1 rs821616	*NDE1* rs4781678	*NDE1* rs2242549	*NDE1* rs881803	*NDE1* rs2075512	*NDE1* Tag Haplotype
**Tramadol**	0.092	0.91	0.67	0.43	0.56	0.95
**Paracetamol**	0.14	0.48	0.66	0.33	0.74	0.43
**Biperiden**	0.53	0.21	0.35	0.41	0.53	0.43
**Chlorpromazine**	0.24	0.39	0.71	0.80	0.77	0.90
**Levomepromazine**	0.35	0.35	0.18	0.28	0.23	0.091
**Perphenazine**	0.18	0.67	0.41	0.37	0.83	0.44
**Thioridazine**	0.87	0.67	0.56	0.21	0.25	0.37
**Haloperidol**	0.096	0.55	0.73	0.61	0.86	0. 60
**Chlorprothixene**	0.40	0.14	0.029	0.036	0.29	0.35
**Zuclopenthixol**	0.11	0.91	0.92	0.14	1.00	na
**Clozapine**	0.83	0.051	0.67	0.93	0.048	0.75
**Olanzapine**	0.81	0.76	0.92	0.85	0.71	0.73
**Quetiapine**	0.87	0.87	0.63	0.23	0.75	0.75
**Risperidone**	0.29	0.067	0.022	0.27	0.17	0.64
**Diazepam**	0.02	0.009	0.007	**0.0015**	0.0078	**0.0016**
**Oxazepam**	0.04	0.49	0.16	0.66	0.26	0.65
**Temazepam**	0.12	0.50	0.91	0.51	0.86	0.69
**Zopiclone**	0.60	0.099	0.031	0.005	0.46	0.11
**Amitriptyline**	0.47	0.96	0.71	0.31	0.076	0.82
**Fluoxetine**	0.27	0.21	0.49	0.51	0.23	0.031
**Citalopram**	0.36	0.0042	**0.0013**	0.055	0.0023	0.026
**Sertraline**	0.92	0.94	0.47	0.19	0.37	0.80
**Mianserin**	0.56	0.055	0.019	0.0023	0.0058	0.0023
**Mirtazapine**	0.64	0.46	0.46	0.82	0.34	0.55

p-values that are below the Bonferroni threshold are in bold.
na = instances where, despite the frequency cut-offs enforced, not enough data points were available for statistical analysis.
Genotype cut-off minor homozygote frequency ≥0.05; Drug usage frequency cut-off = a medication has been used for 3 months or less ≥15 times.

c)

	DISC1 rs821616	*NDE1* rs4781678	*NDE1* rs2242549	*NDE1* rs881803	*NDE1* rs2075512	*NDE1* Tag Haplotype
**Chlorprothixene**	-	-	9.21 (1.26-67.33)	4.94 (1.11-22.05)	-	-
**Clozapine**	-	-	-	-	3.35 (1.01-11.09)	-
**Risperidone**	-	-	0.35 (0.14 - 0.86)	-	-	-
**Diazepam**	3.67 (1.23-10.97)	4.28 (1.44-12.74)	4.28 (1.49-12.31)	**6.20 (2.02-19.09)**	5.14 (1.54-17.17)	**6.13 (1.99-18.85)**
**Oxazepam**	0.26 (0.07-0.94)	-	-	-	-	-
**Zopiclone**	-	-	4.42 (1.15-17.03)	6.07 (1.72-21.42)	-	-
**Fluoxetine**	-	-	-	-	-	0.13 (0.02-0.84)
**Citalopram**	-	0.22 (0.08-0.62)	**0.21 (0.08-0.54)**	-	0.21 (0.07-0.57)	0.33 (0.12-0.88)
**Mianserin**	-	-	7.22 (1.38-37.00)	37.32 (3.62-384.18)	21.78 (2.45-194.05)	115.91 (5.42-2476.98)

Odds ratios that are below the Bonferroni threshold are in bold.

Table S3: Drug names used in this article, their corresponding ATC code and classification, and their main metabolising enzyme(s)

	ATC Code	Use	Class	Metabolising Enzyme(s)[1]
**Tramadol**	N02AX02	Opioids	Other	CYP2D6, CYP3A4
**Paracetamol**	N02BE01	Other analgesics and antipyretics	Anilides	CYP2E1, CYP2A6, CYP3A4, CYP1A2
**Biperiden**	N04AA02	Anticholinergic agents	Tertiary amines	
**Chlorpromazine**	N05AA01	Antipsychotics	Phenothiazines with aliphatic side-chain	CYP2D6
**Levomepromazine**	N05AA02	Antipsychotics	Phenothiazines with aliphatic side-chain	CYP2D6
**Perphenazine**	N05AB03	Antipsychotics	Phenothiazines with piperazine structure	CYP2D6
**Thioridazine**	N05AC02	Antipsychotics	Phenothiazines with piperidine structure	CYP2D6
**Haloperidol**	N05AD01	Antipsychotics	Butyrophenone derivatives	CYP2D6, CYP3A4, CYP3A5, CYP3A7, CYP1A2
**Chlorprothixene**	N05AF03	Antipsychotics	Thioxanthene Derivative	
**Zuclopenthixol**	N05AF05	Antipsychotics	Thioxanthene Derivative	CYP2D6
**Clozapine**	N05AH02	Antipsychotics	Diazepines, oxazepines, thiazepines and oxepines	CYP1A2, CYP3A4
**Olanzapine**	N05AH03	Antipsychotics	Diazepines, oxazepines, thiazepines and oxepines	CYP1A2, CYP2D6
**Quetiapine**	N05AH04	Antipsychotics	Diazepines, oxazepines, thiazepines and oxepines	CYP3A4, CYP3A5, CYP3A7
**Risperidone**	N05AX08	Antipsychotics	Other	CYP2D6, CYP3A4
**Diazepam**	N05BA01	Anxiolytics	Benzodiazepine derivatives	**CYP2C19**, CYP3A4
**Oxazepam**	N05BA04	Anxiolytics	Benzodiazepine derivatives	
**Temazepam**	N05CD07	Hypnotics and Sedatives	Benzodiazepine derivatives	CYP3A4
**Zopiclone**	N05CF01	Hypnotics and Sedatives	Benzodiazepine related drugs	CYP3A4, CYP2C8
**Amitriptyline**	N06AA09	Antidepressants	Non-selective monoamine reuptake inhibitors	**CYP2C19**, CYP2D6, CYP1A2, CYP3A4
**Fluoxetine**	N06AB03	Antidepressants	**Selective serotonin reuptake inhibitors**	CYP2C9, CYP2D6, CYP3A4, **CYP2C19**
**Citalopram**	N06AB04	Antidepressants	**Selective serotonin reuptake inhibitors**	***CYP2C19,*** CYP3A4, CYP2D6
**Paroxetine** [2]	N06AB05	Antidepressants	**Selective serotonin reuptake inhibitors**	CYP2D6
**Sertraline**	N06AB06	Antidepressants	**Selective serotonin reuptake inhibitors**	CYP2B6, **CYP2C19** [3], CYP2C9, CYP3A4
**Fluvoxamine** [2]	N06AB08	Antidepressants	**Selective serotonin reuptake inhibitors**	CYP2D6, CYP1A2
**Escitalopram** [2]	N06AB10	Antidepressants	**Selective serotonin reuptake inhibitors**	***CYP2C19,*** CYP3A4, CYP2D6
**Mianserin**	N06AX03	Antidepressants	Other	CYP3A4, CYP1A2, **CYP2C19**, CYP2D6
**Mirtazapine**	N06AX11	Antidepressants	Other	CYP1A2, CYP2D6, CYP3A4

1 From KEGG Drug database (57), DrugBank (55), PharmaGKB (54), and CPIC Guidelines for SSRIs (56)

2 Medication was not used frequently enough during the 10 years collected here for inclusion in analysis of singular drugs. Drug usage frequency cut-off = a medication has been used for 3 months or less ≥15 times.

3 Expressly stated in the DrugBank database (55) that CYP2C19 only plays a minor role in the metabolism of sertraline. Therefore it has not been grouped with the others that are metabolised by CYP2C19.
